# A review found inadequate reporting of case–control studies of risk factors for pancreatic cancer

**DOI:** 10.1016/j.jclinepi.2020.12.020

**Published:** 2021-05

**Authors:** Angela MacCarthy, Paula Dhiman, Shona Kirtley, Patricia Logullo, Bethan Copsey, Gary S. Collins

**Affiliations:** a Centre for Statistics in Medicine, University of Oxford, Botnar Research Centre, Windmill Road, Oxford OX3 7LD, UK; b NIHR Oxford Biomedical Research Centre, John Radcliffe Hospital, Oxford OX3 9DU, UK

**Keywords:** STROBE, Case–control, Epidemiology, Reporting, Pancreatic cancer, Review

## Abstract

**Objectives:**

Case–control studies are often used to identify the risk factors for pancreatic cancer. The objective of this study was to evaluate the reporting of case–control studies of the risk factors for pancreatic cancer using the Strengthening The Reporting of OBservational Studies in Epidemiology (STROBE) for case–control studies checklist.

**Study Design and Setting:**

We conducted a comprehensive literature search of the MEDLINE and EMBASE databases to identify reports of case–control studies published between 2016 and 2018. We scored article reporting using a reporting adherence form developed from the STROBE checklist for case–control studies, consisting of 14 STROBE items related to the title, abstract, methods, and results sections.

**Results:**

We included reports of 47 case–control studies investigating a variety of risk factors, such as medical conditions and lifestyle factors. Reporting was inconsistent and inadequate. Efforts to address bias and how the study size was arrived at were particularly poorly described. Study cases were described in more detail than study controls.

**Conclusion:**

Reporting of case–control studies remains inadequate more than 10 years after the STROBE reporting guideline was published. Our findings suggest that authors do not understand the extent to which study methods and findings should be reported to enable studies to be fully understood, and their methods reproduced.

## Highlights

• Reporting in a sample of case–control studies was found to be inadequate.
• Clear, complete reporting is essential for study findings to be understood and compared.
• Clearly described methods allow studies to be reproduced.
• The use of the Strengthening The Reporting of OBservational Studies in Epidemiology reporting guideline is essential when reporting observational studies.

**What is new?**

• Reporting in a sample of case-control studies was found to be inadequate.
• Clear, complete reporting is essential for study findings to be understood and compared.
• Clearly described methods allow studies to be reproduced.
• The use of the ‘Strengthening The Reporting of OBservational Studies in Epidemiology’ (STROBE) reporting guideline is essential when reporting observational studies.

## 1 Introduction

The incidence of pancreatic cancer is rising worldwide. The 5-year survival rate is only 9%, as it is often diagnosed at an advanced stage [1]. Many studies have attempted to identify modifiable risk factors for this cancer in numerous settings. The risk factors that have been investigated include medical conditions (diabetes, pancreatitis, infections, and asthma), medication use (proton-pump inhibitors, statins, and aspirin), those related to lifestyle (diet, cigarette smoking, coffee, and alcohol), and other factors (obesity, blood group, microbiome, menstrual, and reproductive factors). Despite so many published studies, risk factors are still so poorly characterized that they cannot be used to develop preventative measures [2].

Pancreatic cancer risk factor research is often informed by studies using a case–control design, which is prone to systemic biases and confounding. Such studies need clear and complete reporting to ensure they can contribute toward establishing evidence-based risk factors. In general, research should be published in sufficient detail for the methods to be evaluated or reproduced and the findings to be compared between studies and summarized in reviews. Many reviews of reporting have found that observational health research is routinely poorly reported in all studied medical fields, including oncology [3]. The quality of reporting of case–control studies in oncology is unclear, and no study has examined the reporting of case–control studies in pancreatic cancer.

Our primary objective in this study was to assess the completeness of reporting in the title, abstract, methods, and results sections in a sample of recently published case–control studies, using the Strengthening The Reporting of OBservational Studies in Epidemiology (STROBE) checklist for case–control studies [4]. We also examined the extent to which STROBE items were reported, but in insufficient detail to enable the study methods to be reproduced.

## 2 Materials and methods

### 2.1 Literature search

We conducted a comprehensive literature search on July 31, 2019, to identify a sample of recently published case–control studies on pancreatic cancer. We searched the MEDLINE and EMBASE databases via the OVID platform for relevant references published between January 1, 2016, and December 31, 2018. The search strategies were developed by an experienced information specialist (S.K.) and underwent iterative testing on each database searched. Search terms included MeSH and EMTREE headings and free-text terms for “pancreatic cancer,” study type terms related to “case–control studies,” and observational research–related and statistical-related terms (e.g., “odds ratios,” “risk,” and “match”). Except for the date limits mentioned previously, no language or other limits were applied to the search. The full search strategies for MEDLINE and EMBASE are presented in Appendix A.

Owing to the difficulties in reliably searching for observational studies in bibliographic databases and the variation in terminology used by researchers, we deliberately included a broad range of search terms to identify case–control studies. We aimed to evaluate 30–50 case–control studies to obtain an overview of reporting quality. We therefore searched both databases from 2016 to 2018 to ensure that we retrieved enough eligible case–control studies.

All references retrieved from EMBASE and MEDLINE were imported into EndNote (version X7.8) (Clarivate, Philadelphia, PA, USA). References were deduplicated using the “find duplicates” facility in EndNote. Remaining citations were checked manually for duplicate records that were missed by the electronic deduplication process. The titles, abstracts, and full texts (when necessary) of all references were evaluated by one author (A.M.).

### 2.2 Exclusion criteria

We excluded articles from the results of the search if they were (1) duplicate citations (found as both MEDLINE and EMBASE were searched), (2) not published in English, (3) not about pancreatic cancer or they included other cancer diagnostic groups with pancreatic cancer, or (4) not case–control studies. Reports of case–control studies of genetic associations, genetic polymorphisms, and molecular epidemiology were excluded because these articles should be written with guidance from the STrengthening the REporting of Genetic Association Studies [5] or STROBE-ME [6] reporting guidelines. A second author (P.L.) read all the case–control studies to confirm that they met the inclusion criteria: complete reports of case–control studies (not study reports summarized as letters, for example), suitable to be scored for adherence to the STROBE checklist for case–control studies.

### 2.3 Development of the reporting adherence form

We used the STROBE checklist for case–control studies [4], consisting of 22 reporting items, as a starting point to develop a reporting adherence form. We included 14 STROBE checklist for case–control studies items in the form, referring to the title and abstract (Item 1), methods (Items 4–12), and results (Items 13–16) sections. We excluded the remaining eight STROBE items that referred to the introduction (Items 2 and 3), discussion (Items 18–21), and other information (Item 22), as these items are less relevant to reproducibility. We also excluded item 17, “Other analyses,” as this item may not have been relevant to all the articles in our study.

We expanded the items into “subitems,” as many of the STROBE checklist for case–control studies items each consist of multiple pieces of information that should be reported. Each subitem therefore referred to one discrete piece of information about a study. For example, STROBE Item 6 about participants reads: “(a) Give the eligibility criteria, and the sources and methods of case ascertainment and control selection. G. the rationale for the choice of cases and controls.” We expanded this item into 10 subitems relating to cases (subitems 6.1, 6.3, 6.5, 6.7), controls (subitems 6.2, 6.4, 6.6, 6.8), the matching criteria (6.9), and number of controls per case (6.10). Three of the 14 items (Items 4, 9, and 10) were not split into subitems and were converted into one question each.

We excluded two parts of STROBE items that related to cohort studies, that is, “periods of follow-up” in STROBE Item 5 and “completing follow-up” in STROBE Item 13. We also excluded four parts of STROBE items that were not relevant to all the studies in our sample, such as “subgroups and interactions” and “sensitivity analyses” in STROBE item 12. Appendix B lists the eight excluded STROBE items and all excluded parts of STROBE items. We were left with 65 STROBE subitems to evaluate.

To evaluate reporting completeness, we converted each remaining STROBE subitem into a question, resulting in a 65-question reporting adherence form. Four reviewers (A.M., B.C., P.D., and P.L.) piloted the form on five case–control articles that were to be included in the review. The final reporting adherence form, after minor amendments to the text guided by the pilot, is shown in Appendix C.

### 2.4 Evaluation of reporting–scoring the STROBE subitems

Each article was evaluated by a statistician (B.C. or P.D.) and a nonstatistician (A.M. or P.L.). Articles were allocated to pairs of evaluators from an alphabetical list of first authors. All evaluations were carried out “blind,” without sight of the score allocated by the other evaluator. Evaluators received the reporting adherence form (Appendix C), a copy of the STROBE Explanation and Elaboration paper [7], and project-specific guidance (Appendix D). The latter summarized information from the STROBE Explanation and Elaboration paper.

We evaluated reporting in the articles by producing two scores:

• We scored at the subitem level to produce the STROBE subitem score.
• We then examined reporting completeness by combining the STROBE subitem scores to produce the full STROBE item score.

To calculate the STROBE subitem score, each of the 65 questions on the adherence form was scored as follows:

• Reported in sufficient detail to be reproduced
• Reported, but not in sufficient detail to be reproduced
• Not reported at all
• Unsure
• Not applicable

Scoring conflicts between evaluators were resolved by face-to-face discussion. If both evaluators scored a subitem “unsure,” the subitem was scored “not reported at all.”

The full STROBE item score was assigned as follows:

• An STROBE item was considered fully reported when all subitems were “reported in sufficient detail to be reproduced.”
• An STROBE item was considered partly reported when at least one but not all subitems were “reported in sufficient detail to be reproduced.”
• An STROBE item was considered not reported when all subitems were either “not reported” or “reported but not in sufficient detail to be reproduced.”

### 2.5 Data analysis

The data were imported into STATA version 15 (StataCorp, College Station, TX, USA) and analyzed by a statistician (P.D.). The primary outcome was completeness of reporting at the STROBE item level. Reporting data were summarized at the STROBE item and subitem levels. Descriptive statistics (number and percentages), graphs, and a narrative synthesis are used to describe reporting completeness and overall reporting for each article.

## 3 Results

We identified and evaluated 47 articles reporting case–control studies that examined potential risk factors for pancreatic cancer. The details of these studies are shown in Appendix E.

**Fig. 1:**
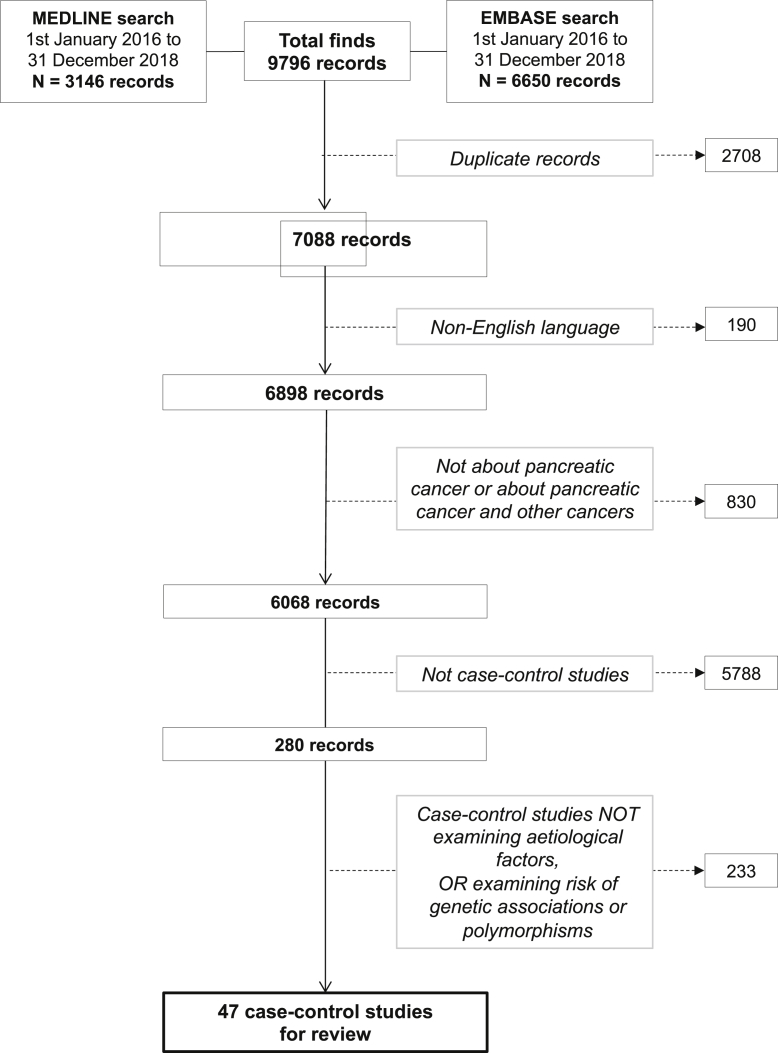
Flow diagram showing the process of selection of articles for this review.

The flow diagram (Fig. 1) shows how the articles were selected.

### 3.1 STROBE item scores

**Fig. 2:**
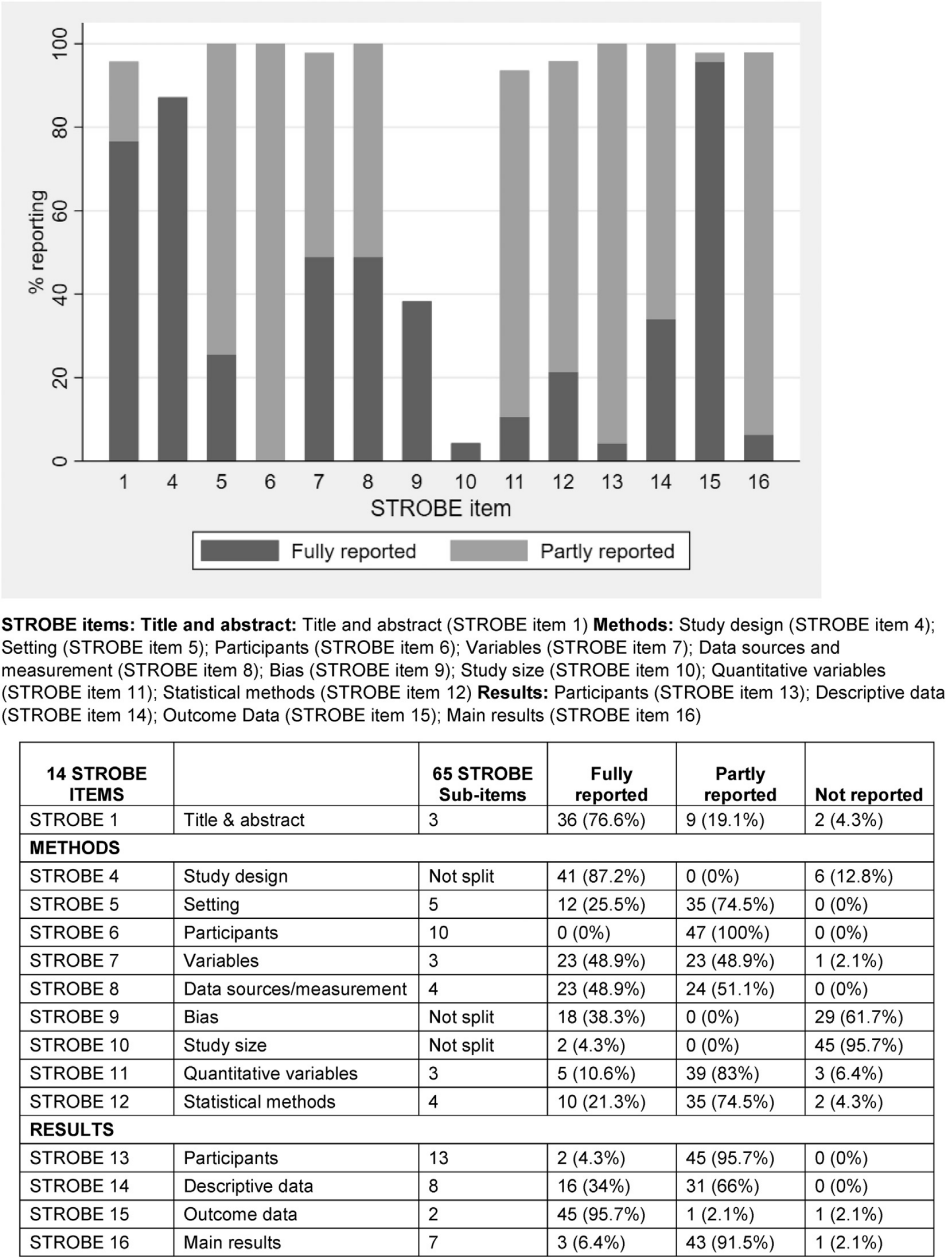
Completeness of reporting of 14 STROBE items in 47 articles describing case–control studies.

Fig. 2 illustrates how completely the 14 full STROBE items were reported. No item was fully reported in all articles, with the percentage of articles fully reporting an item ranging from 0% for details of participants in the methods (Item 6) to 95.7% for reporting outcome data in the results section (Item 15). The title and abstract (Item 1) and study design (Item 4) were both generally reported well, by 76.6% and 87.2% of articles, respectively. Some of the items that were only fully reported in a small number of articles or not fully reported at all were at least partly reported by the remaining articles, such as those relating to participants (Items 6 and 13) and descriptive data (Item 14). However, efforts to address bias (Item 9) and details of how the study size was arrived at (Item 10) were both very poorly reported, with 61.7% and 95.7% of articles respectively not reporting these items at all.

### 3.2 STROBE subitem score

Table 1, Table 2, Table 3 show the percentage of the STROBE subitems reported in the 47 evaluated articles. Reporting completeness varied considerably, from only two articles (4.3%) describing in sufficient detail how the study size was arrived at (Item 10) to all articles (100%) describing the source of study cases (subitem 6.3; Table 2).

**Table 1:** Reporting of STROBE subitems relating to the study title and abstract in 47 articles describing case–control studies

STROBE Item 1 title and abstract (three subitems)	No (%) of articles that reported subitem in sufficient detail	No (%) of articles that reported subitem but not in sufficient detail	No (%) of articles that did not report the subitem at all	Not applicable
1.1 Indicate the study's design with a commonly used term in the title or the abstract	41 (87.2)	3 (6.4)	3 (6.4)	0 (0)
1.2 Provide in the abstract an informative and balanced summary of what was done	38 (80.9)	8 (17)	1 (2.1)	0 (0)
1.3 Provide in the abstract an informative and balanced summary of what was found	44 (93.6)	3 (6.4)	0 (0)	0 (0)

**Table 2:** Reporting of STROBE subitems relating to the study methods in 47 articles describing case–control studies

STROBE subitem	No (%) of articles that reported subitem in sufficient detail	No (%) of articles that reported subitem but not in sufficient detail	No (%) of articles that did not report the subitem at all	Not applicable
STROBE Item 4 Study design				
4 Present key elements of study design early in the paper	41 (87.2)	4 (8.5)	2 (4.3)	0 (0)
STROBE Item 5 Study setting (five subitems)				
5.1 Describe the study setting	42 (89.4)	3 (6.4)	2 (4.3)	0 (0)
5.2 Describe the study locations	43 (91.5)	4 (8.5)	0 (0)	0 (0)
5.3 The period of recruitment	39 (83)	4 (8.5)	4 (8.5)	0 (0%)
5.4 Describe the period of exposure	14 (29.8)	4 (8.5)	22 (46.8)	7 (14.9)
5.5 Describe the period of data collection	25 (53.2)	6 (12.8)	16 (34)	0 (0)
STROBE Item 6 Participants (10 subitems)				
6.1 Give the eligibility criteria for cases	39 (83)	5 (10.6)	3 (6.4)	0 (0)
6.2 Give the eligibility criteria for controls	35 (74.5)	1 (2.1)	11 (23.4)	0 (0)
6.3 Give the source of cases	47 (100)	0 (0)	0 (0)	0 (0)
6.4 Give the source of controls	45 (95.7)	0 (0)	2 (4.3)	0 (0)
6.5 Give the methods of case ascertainment	41 (87.2)	1 (2.1)	5 (10.6)	0 (0)
6.6 Give the methods of control selection	40 (85.1)	0 (0)	7 (14.9)	0 (0)
6.7 Give the rationale for the choice of cases	13 (27.7)	0 (0)	34 (72.3)	0 (0)
6.8 Give the rationale for the choice of controls	13 (27.7)	0 (0)	34 (72.3)	0 (0)
6.9 Give the matching criteria	35 (74.5)	3 (6.4)	7 (14.9)	2 (4.3)
6.10 Give the number of controls per case	37 (78.7)	6 (12.8)	4 (8.5)	0 (0)
STROBE Item 7 Variables (three subitems)				
7.1 Clearly define all the outcomes	36 (76.6)	10 (21.3)	1 (2.1)	0 (0)
7.2 Clearly define all exposures evaluated	36 (76.6)	9 (19.1)	2 (4.3)	0 (0)
7.3 Clearly define all potential confounders	35 (74.5)	11 (23.4)	1 (2.1)	0 (0)
STROBE item 8 Data sources/measurement (four subitems) for each variable of interest				
8.1 Give sources of data for cases	47 (100)	0 (0)	0 (0)	0 (0)
8.2 Give sources of data for controls	45 (95.7)	1 (2.1)	1 (2.1)	0 (0)
8.3 Give details of methods of assessment (measurement) for cases	26 (55.3)	18 (38.3)	3 (6.4)	0 (0)
8.4 Give details of methods of assessment (measurement) for controls	36 (76.6)	7 (14.9)	4 (8.5)	0 (0)
STROBE Item 9 Bias				
9 Describe any efforts to address potential sources of bias	18 (38.3)	3 (6.4)	26 (55.3)	0 (0)
STROBE Item 10 Study size				
10 Explain how the study size was arrived at	2 (4.3)	2 (4.3)	43 (91.5)	0 (0)
STROBE Item 11 Quantitative variables (three subitems)				
11.1 Explain how quantitative variables were handled in the analyses	33 (70.2)	7 (14.9)	7 (14.9)	0 (0)
11.2 Which groupings were chosen	41 (87.2)	4 (8.5)	2 (4.3)	0 (0)
11.3 Why groupings were chosen	7 (14.9)	2 (4.3)	38 (80.9)	0 (0)

**Table 3:** Reporting of STROBE subitems relating to the study results in 47 articles describing case–control studies

STROBE subitem	No (%) of articles that reported subitem in sufficient detail	No (%) of articles that reported subitem but not in sufficient detail	No (%) of articles that did report the subitem at all	Not applicable
STROBE Item 13 Participants (13 subitems)				
13.1 Report numbers potentially eligible—cases	22 (46.8)	1 (2.1)	24 (51.1)	0 (0)
13.2 Report numbers potentially eligible—controls	15 (31.9)	1 (2.1)	31 (66)	0 (0)
13.3 Report numbers examined for eligibility—cases	22 (46.8)	1 (2.1)	24 (51.1)	0 (0)
13.4 Report numbers examined for eligibility—controls	13 (27.7)	3 (6.4)	31 (66)	0 (0)
13.5 Report numbers confirmed eligible—cases	28 (59.6)	0 (0)	19 (40.4)	0 (0)
13.6 Report numbers confirmed eligible—controls	17 (36.2)	0 (0)	30 (63.8)	0 (0)
13.7 Report numbers included in the study—cases	36 (76.6)	0 (0)	11 (23.4)	0 (0)
13.8 Report numbers included in the study—controls	28 (59.6)	0 (0)	19 (40.4)	0 (0)
13.9 Report numbers analyzed—cases	47 (100)	0 (0)	0 (0)	0 (0)
13.10 Report numbers analyzed—controls	47 (100)	0 (0)	0 (0)	0 (0)
13.11 Give reasons for nonparticipation at each stage—cases	15 (31.9)	3 (6.4)	29 (61.7)	0 (0)
13.12 Give reasons for nonparticipation at each stage—controls	9 (19.1)	3 (6.4)	35 (74.5)	0 (0)
13.13 Consider use of a flow diagram	4 (8.5)	4 (8.5)	39 (83)	0 (0)
STROBE Item 14 Descriptive data (eight subitems)				
14.1 Give demographic, clinical, social details—cases	46 (97.9)	1 (2.1)	0 (0)	0 (0)
14.2 Give demographic, clinical, social details—controls	46 (97.9)	0 (0)	1 (2.1)	0 (0)
14.3 Give information on exposures—cases	44 (93.6)	1 (2.1)	2 (4.3)	0 (0)
14.4 Information on exposures—controls	45 (95.7)	0 (0)	2 (4.3)	0 (0)
14.5 Give information on potential confounders—cases	39 (83)	6 (12.8)	2 (4.3)	0 (0)
14.6 Information on potential confounders—controls	35 (74.5)	9 (19.1)	3 (6.4)	0 (0)
14.7 Indicate number of participants with missing data for each variable of interest—cases	20 (42.6)	5 (10.6)	22 (46.8)	0 (0)
14.8 Indicate number of participants with missing data for each variable of interest—controls	18 (38.3)	6 (12.8)	23 (48.9)	0 (0)
STROBE Item 15 Outcome data (two subitems)				
15.1 Report numbers in each exposure category or summary measures of exposure—cases	45 (95.7)	0 (0)	2 (4.3)	0 (0)
15.2 Report numbers in each exposure category or summary measures of exposure—controls	46 (97.9)	0 (0)	1 (2.1)	0 (0)
STROBE Item 16 Main results (seven subitems)				
16.1 Give unadjusted estimates	19 (40.4)	1 (2.1)	27 (57.4)	0 (0)
16.2 Give precision for unadjusted estimates	19 (40.4)	1 (2.1)	27 (57.4)	0 (0)
16.3 Give confounder-adjusted estimates	45 (95.7)	1 (2.1)	1 (2.1)	0 (0)
16.4 Give precision for confounder-adjusted estimates	46 (97.9)	0 (0)	1 (2.1)	0 (0)
16.5 Make clear which confounders were adjusted for	45 (95.7)	0 (0)	2 (4.3)	0 (0)
16.6 Make clear why confounders were included	4 (8.5)	1 (2.1)	42 (89.4)	0 (0)
16.7 Report category boundaries when continuous variables were categorized	38 (80.9)	5 (10.6)	2 (4.3)	2 (4.3)

### 3.3 Study setting

Three of the five subitems relating to the study setting—the study setting (subitem 5.1), location (subitem 5.2), and recruitment period (subitem 5.3)—were all sufficiently reported in more than 80% of the articles. However, the exposure (subitem 5.4) and data collection (subitem 5.5) periods were only sufficiently reported in 29.8% and 53.2% of articles, respectively. The exposure period was considered by the evaluators to not be applicable to 14.9% of the studies (Table 2).

### 3.4 Confounding

Confounding was generally well described, with 74.5% of articles sufficiently defining potential confounders (subitem 7.3) and 83% of articles sufficiently describing the methods used to control for confounding (subitem 12.2; Table 2).

Most articles sufficiently presented confounder-adjusted estimates of risk (95.7%, subitem 16.3) and their precision (97.7%, subitem 16.4). Although nearly all (95.7%) the articles sufficiently described which confounders were adjusted for in the results (subitem 16.5), only four articles (8.5%) explained why these variables had been selected as possible confounding variables (subitem 16.6). More articles described potential confounders for cases (83%, subitem 14.5) than controls (74.5%, subitem 14.6; Table 3).

### 3.5 Bias

Only 18 articles (38.3%) sufficiently described the methods used to address potential sources of bias (Item 9). Another three articles (6.4%) reported this item in insufficient detail. Most articles (26, 55.3%) did not report the item (Table 2).

### 3.6 Study size

Only two articles (4.3%) sufficiently described how the study size was arrived at (Item 10). Most of the articles (91.5%) did not describe this essential item at all (Table 2).

### 3.7 Study participants

All the articles (100%) sufficiently described the source of cases in the methods section (subitem 6.3) and nearly all (95.7%) the source of controls (subitem 6.4). However, the rationale for the choice of cases (subitem 6.7) and controls (subitem 6.8) was only reported in sufficient detail in 27.7% of articles. There was not much difference in how well the methods of ascertainment were described for cases (87.2%, subitem 6.5) and the methods of selection for controls (85.1%, subitem 6.6). Eligibility criteria were sufficiently described for cases (83%, subitem 6.1) more often than for controls (74.5%, subitem 6.2; Table 2).

STROBE Item 13 (Table 3) explains how to describe the flow of cases and controls through the study in the results section. The numbers of cases and controls potentially eligible, examined for eligibility, and confirmed eligible were poorly reported in the results section (subitems 13.1–13.6). Numbers for controls were more poorly reported (subitems 13.2, 13.4, and 13.6) than cases (subitems 13.1, 13.3, and 13.5). More articles reported how many cases were included in the study (76.6%, subitem 13.7) than controls (59.6%, subitem 13.8). However, all articles reported the numbers of cases and controls in the statistical analysis (subitems 13.9 and 13.10).

The reasons for nonparticipation at each stage of the study (Table 3) were particularly poorly reported for both cases (31.9%, subitem 13.11) and controls (19.1%, subitem 13.12). Only four articles (8.5%) gave a complete flow diagram (subitem 13.13), whereas another four articles (8.5%) provided a flow diagram for study cases only or provided a diagram in supplementary material.

The demographic, clinical, and social characteristics of cases and controls (Table 3; subitems 14.1 and 14.2) and their exposures (subitems 14.3 and 14.4) were sufficiently reported by more than 90% of articles. Fewer articles sufficiently reported potential confounders for cases (83%, subitem 14.5) than for controls (74.5%, subitem 14.6).

### 3.8 Statistical methods

Information about missing data was generally poorly reported. Only 34% of articles sufficiently described how missing data were addressed in the statistical methods (subitem 12.3; Table 2). The extent of missing data was slightly better reported, with 42.6% of articles reporting how much data were missing for cases (subitem 14.7) and 38.3% of articles for controls (subitem 14.8; Table 3).

### 3.9 Outcome data

Nearly all the articles sufficiently reported the number of cases (95.7%, subitem 15.1) and controls (97.9%, subitem 15.2) in each exposure category in the results section (Table 3).

## 4 Discussion

Overall, the articles in our review inconsistently and inadequately reported the methods and results from case–control studies. The reporting of bias, missing data, how the study size was arrived at, and details of statistical methods used were particularly poor.

Most articles reported the study design in the title or abstract using a commonly used term. This result is encouraging, as the title and abstract may be the only parts of the article that are read. As the abstract and title are also used as search fields in electronic literature databases such as MEDLINE or EMBASE, better reporting of these items will also allow articles to be retrieved in literature searches.

Except for study design (Item 4), most of the methods items were fully reported in less than 50% of the articles. Statistical methods (Item 12) were particularly poorly reported, with only 21% of articles fully reporting this item. Missing data, for example, can be handled with a variety of techniques. Although some studies added a footnote in their tables reporting the number of cases with missing data, they did not describe how they handled these missing data.

Study methods were often better reported than the rationale for those methods. For example, the source of cases and controls (subitems 6.3 and 6.4) was well reported by almost all the included articles, but why these sources were chosen (subitems 6.7 and 6.8) was only described in just more than one-fourth of the articles. Similarly, the description of *which* groupings of quantitative variables were chosen (subitem 11.2) was far better reported than *why* the groupings were chosen (subitem 11.3). Research articles are read by a much wider group of people than experienced researchers: early career investigators, students, and, increasingly, the public. Clear and complete information about why study methods were chosen will enhance readers’ understanding of the study findings.

Our results are in general agreement with an earlier study of reporting in 38 case–control studies [8]. That study also found variable reporting quality and that certain methods and results items were inadequately reported. However, the reviewed articles were published in only one medical journal and were mostly published before the STROBE guidance was published in 2007 [9]. We did not restrict our literature search to any particular journal and evaluated articles published a decade after STROBE.

Despite the longstanding existence of STROBE guidance, the authors of our reviewed articles may not have used it. In fact, only one article stated that the authors used STROBE to guide their reporting. Although this is not evidence of nonusage in the other studies, it is not good news from the perspective of good reporting. Alternatively, the authors may have used STROBE but still failed to report study details. This might have been because authors were not convinced that it was necessary to report the detail within each STROBE item. Or perhaps authors were insufficiently experienced in the use of reporting guidelines (possibly due to a lack of training) or because the information in the reporting guideline was not clear enough.

A recent online survey about experiences with, and attitudes toward, the STROBE Statement [10] indicated that some respondents were still unaware that this guidance existed. Others were aware of it but failed to use it because they did not appreciate the implications of using it or found it difficult to use. The authors of the survey concluded that there is a need for better communication about the importance of using reporting guidelines, particularly how their use improves study reproducibility. They also concluded that a change in culture will be required to improve the uptake of such guidance. Emphasis on the importance of reporting guidelines and the issue of reproducibility in postgraduate, early career, and professional development educational schemes is essential.

A published research article is only useful to readers if it can be readily understood and contains enough information for the reader to assess the methods and reliability of the findings. Unfortunately, as we found here, much research is published with insufficient detail for studies to be reproduced or to be useful to other researchers when planning new research projects.

Once a research report is published, it takes on a permanent existence in the scientific literature, so there are long-term consequences to poor reporting: clinicians cannot use a poorly described piece of research to inform their practice, and reliable preventative advice for medical conditions cannot be developed if reported findings cannot be compared and summarized. Articles that do not contain sufficient details of methods and findings may need to be excluded from systematic reviews and meta-analyses, biasing the resulting review findings. Inadequate reporting thus leads to the waste of precious research resources and impedes scientific progress.

Although numerous validated reporting guidelines exist to help ensure adequate reporting, authors are either not aware of them or for other unknown reasons are failing to use them. Journals can address this issue by insisting on their use as part of the submission process and by checking that they have been used. Reporting guidelines are highlighted by the International Committee of Medical Journal Editors in their guidance on article preparation (http://www.icmje.org/recommendations/browse/manuscript-preparation/preparing-for-submission.html), and the STROBE Statement has been translated into Chinese, German, Greek, Japanese, Portuguese (unofficial translation), Spanish, and Italian. Guidance on how to integrate reporting guidelines into the workflow of journals has recently been developed and is now available on the EQUATOR Network website (https://www.equator-network.org/toolkits/using-guidelines-in-journals/). Clear, complete reporting and reference to reporting guidelines will also help journal editors and peer-reviewers.

Our results point to the need for improved promotion of and training in the use of STROBE guidance and an explanation of its importance. We believe that the STROBE reporting guideline should be reviewed, clarified, and updated, as has happened with other major reporting guidelines such as CONSORT [11]. Any updated version of STROBE should be accompanied by the publication of a reporting adherence evaluation tool.

A strength of our study was that we designed a reporting adherence form, which explicitly listed each piece of information within a STROBE item as a subitem; consequently, we were able to score whether each component appeared in the article. For example, we scored whether information appeared for cases and controls separately, when appropriate. We did not record subitems as simply present or absent but included an intermediate category where subitems were mentioned but in insufficient detail for reproducibility. We also used four evaluators to score reporting. Two of the evaluators were statisticians with a good understanding of what should be reported for statistical methods, and their expertise enhanced our discussions when resolving scoring conflicts.

A limitation of our study was that our reporting adherence form was based on a reporting guideline. Such guidelines are not explicitly designed to be used to evaluate reporting quality, which causes problems when turning them into tools to assess reporting [12]. We feel that it would be helpful if reporting guideline developers produced a tool to measure adherence to their guideline for use when evaluating reporting. Heus et al. [13] have done so for the Transparent Reporting of a multivariable prediction model for Individual Prognosis Or Diagnosis statement [14].

## 5 Conclusion

We found numerous examples of inadequate reporting in a sample of recently published case–control studies investigating risk factors for pancreatic cancer. Researchers should use reporting guidelines to help them to report all essential information about their study to ensure that the methods are reproducible and that the results can be used in clinical practice or to inform further research. To improve support for researchers, the STROBE reporting guideline should be updated and promoted, and an accompanying reporting adherence evaluation tool created.

## Supplementary data

Data Profile
