# Supplementary material for: A review found inadequate reporting of case–control studies of risk factors for pancreatic cancer
Source: J Clin Epidemiol. 2021 May;133:32–42. doi: 10.1016/j.jclinepi.2020.12.020 (PMC8168827; doi:10.1016/j.jclinepi.2020.12.020)
Supplement: Appendix A [file mmc2.docx]

**Appendix A**

Search strategies for pancreatic cancer case-control studies: EMBASE and Medline

**MEDLINE**

Database & platform: Medline (Ovid MEDLINE® Epub Ahead of Print, In-Process & Other Non-Indexed Citations, Ovid MEDLINE® Daily and Ovid MEDLINE®) 1946 to present (via OVID)

Search date: 31 July 2019

1. Pancreatic Neoplasms/ep [Epidemiology]

2. Pancreatic Neoplasms/et [Etiology]

3. Carcinoma, Pancreatic Ductal/ep [Epidemiology]

4. Carcinoma, Pancreatic Ductal/et [Etiology]

5. ((pancreas or pancreatic or acinar) adj3 (cancer$ or tumour$ or tumor$ or carcinoma$ or malignan$ or neoplas$ or sarcoma$ or adenocarcinoma$ or carcinogen$)).ti,kw.

6. (pancreatoblastoma or PDAC or PNETs or PNENs).ti,kw.

7. ((pancreas or pancreatic) adj2 pseudopapillary adj2 neoplasm$).ti,kw.

8. ("branch duct intraductal papillary mucinous neoplasm" or "branch duct intraductal papillary mucinous neoplasms" or "BD-IPMN" or "BD-IPMNs" or "IPMN" or "IPMNs" or "intraductal papillary mucinous neoplasm" or "intraductal papillary mucinous neoplasms").ti,kw.

9. or/1-8

10. Case-Control Studies/

11. Retrospective Studies/

12. Prospective Studies/

13. Odds Ratios/

14. "odds ratio$".ab.

15. regression.ti,ab,kw.

16. ((patient$ or participant$ or subject$) adj4 control$).ti,ab,kw.

17. (case adj2 control adj1 (stud$ or analys$ or design$)).ti,ab,kw.

18. (case$ adj5 control$).ti,ab,kw.

19. ((retrospective or prospective or epidemiolo$ or aetiolo$ or etiolo$) adj1 (stud$ or analys$ or design$ or evaluation$)).ti,ab,kw.

20. Risk Factors/

21. Risk/

22. Observational Study/

23. (observational adj1 stud$).ti,ab,kw.

24. (retrospective adj1 chart adj1 review).ti,ab,kw.

25. risk.ti,ab,kw.

26. (match$ or unmatch$).ti,ab,kw.

27. ("age-match$" or "sex-match$").ti,ab,kw.

28. or/10-27

29. 9 and 28

30. limit 29 to yr ="2016-2018"

**EMBASE**

Database & platform: Embase 1974 to present (via OVID)

Search date: 31 July 2019

1. Pancreas cancer/ep, et [Epidemiology, Etiology]

2. Pancreas tumor/ep, et [Epidemiology, Etiology]

3. Pancreas carcinoma/ep, et [Epidemiology, Etiology]

4. Pancreas metastasis/ep, et [Epidemiology, Etiology]

5. Intraductal papillary mucinous tumor/ep, et [Epidemiology, Etiology]

6. Mucinous cystic neoplasm/ep, et [Epidemiology, Etiology]

7. Pancreas adenoma/ep, et [Epidemiology, Etiology]

8. Pancreas islet cell tumor/ep, et [Epidemiology, Etiology]

9. Pancreatic incidentaloma/ep [Epidemiology]

10. Solid pseudopapillary tumor/

11. Pancreas adenocarcinoma/ep, et [Epidemiology, Etiology]

12. ((pancreas or pancreatic or acinar) adj3 (cancer$ or tumour$ or tumor$ or carcinoma$ or malignan$ or neoplas$ or sarcoma$ or adenocarcinoma$ or carcinogen$)).ti,kw.

13. (pancreatoblastoma or PDAC or PNETs or PNENs).ti,kw.

14. ((pancreas or pancreatic) adj2 pseudopapillary adj2 neoplasm$).ti,kw.

15. ("branch duct intraductal papillary mucinous neoplasm" or "branch duct intraductal papillary mucinous neoplasms" or "BD-IPMN" or "BD-IPMNs" or "IPMN" or "IPMNs" or "intraductal papillary mucinous neoplasm" or "intraductal papillary mucinous neoplasms").ti,kw.

16. or/1-15

17. Case-Control Study/

18. Retrospective Study/

19. Prospective Study/

20. Odds Ratio/

21. "odds ratio$".ab.

22. regression.ti,ab,kw.

23. ((patient$ or participant$ or subject$) adj4 control$).ti,ab,kw.

24. (case adj2 control adj1 (stud$ or analys$ or design$)).ti,ab,kw.

25. (case$ adj5 control$).ti,ab,kw.

26. ((retrospective or prospective or epidemiolo$ or aetiolo$ or etiolo$) adj1 (stud$ or analys$ or design$ or evaluation$)).ti,ab,kw.

27. Risk Factor/

28. Risk/

29. Observational Study/

30. (observational adj1 stud$).ti,ab,kw.

31. (retrospective adj1 chart adj1 review).ti,ab,kw.

32. risk.ti,ab,kw.

33. (match$ or unmatch$).ti,ab,kw.

34. ("age-match$" or "sex-match$").ti,ab,kw.

35. or/17-34

36. 16 and 35

37. limit 36 to yr ="2016-2018"
