# Supplementary material for: A review found inadequate reporting of case–control studies of risk factors for pancreatic cancer
Source: J Clin Epidemiol. 2021 May;133:32–42. doi: 10.1016/j.jclinepi.2020.12.020 (PMC8168827; doi:10.1016/j.jclinepi.2020.12.020)
Supplement: Appendix B [file mmc3.docx]

**Appendix B: STROBE for case-control studies checklist Items (and sub-items) excluded from our reporting adherence form**

**Note: 2 Pages**

**8 complete Items** from the STROBE for case-control checklist that were excluded from our reporting adherence form because 7 were not relevant for reproducibility and 1 was not relevant to all articles in our sample (i.e. Item 17)

From INTRODUCTION

STROBE Item 2 Background/rationale: Explain the scientific background and rationale for the investigation being reported

STROBE Item 3 Objectives: State specific objectives, including any prespecified hypotheses

**From RESULTS**

STROBE Item 17 Other analyses: Report other analyses done - e.g. analyses of subgroups and interactions, and sensitivity analyses

**From DISCUSSION**

STROBE Item 18 Key results: Summarise key results with reference to study objectives

STROBE Item 19 Limitations: Discuss limitations of the study, taking into account sources of potential bias or imprecision. Discuss both direction and magnitude of any potential bias

STROBE Item 20 Interpretation: Give a cautious overall interpretation of results considering objectives, limitations, multiplicity of analyses, results from similar studies, and other relevant evidence

STROBE Item 21 Generalisability: Discuss the generalisability (external validity) of the study results

**From OTHER INFORMATION**

STROBE item 22 Funding: Give the source of funding and the role of the funders for the present study and, if applicable, for the original study on which the present article is based

**Parts of STROBE sub-items** that were excluded from our reporting evaluation form

**METHODS**

**STROBE Item 5 Setting:**

Describe the setting, locations, and relevant dates, including periods of recruitment, exposure, follow-up, and data collection

From this item we only excluded *‘period of follow-up’* not relevant for reporting case-control studies

**STROBE Item 7** **Variables:**

Clearly define all outcomes, exposures, predictors, potential confounders, and effect

modifiers. Give diagnostic criteria, if applicable.

From this item we only excluded *‘effect modifiers’* and *‘diagnostic criteria’* for outcomes, exposures and confounders as these may not have been relevant in all studies in our sample

**STROBE Item 8 Data sources/measurement**

For each variable of interest, give sources of data and details of methods of assessment (measurement). Describe comparability of assessment methods if there is more than one group

From this item we only excluded *‘comparability of assessment methods’* as this may not have been relevant in all studies in our sample

**STROBE Item 12 Statistical** **methods**

(a)Describe all statistical methods, including those used to control for confounding

(*b*) Describe any methods used to examine subgroups and interactions

(*c*) Explain how missing data were addressed

(*d*) If applicable, explain how matching of cases and controls was addressed

(*e*) Describe any sensitivity analyses

From this item we only excluded (b) *‘methods used to examine* *subgroups’* and *‘interactions’* and *‘sensitivity analyses’* as these may not have been relevant in all studies in our sample

**RESULTS**

**STROBE Item 13 Participants:**

(a) Report numbers of individuals at each stage of study— e.g. numbers potentially eligible, examined for eligibility, confirmed eligible, included in the study, completing follow-up, and analysed

(b) Give reasons for non-participation at each stage

(c) Consider use of a flow diagram

From this item we only excluded *‘numbers completing follow up’* – not relevant for reporting case-control studies

**STROBE Item 16 Main results:**

Give unadjusted estimates and, if applicable, confounder-adjusted estimates and their precision (e.g. 95% confidence interval). Make clear which confounders were adjusted for and why they were included

Report category boundaries when continuous variables were categorized

If relevant, consider translating estimates of relative risk into absolute risk for a meaningful time period

From this item we only excluded *‘category boundaries* and *‘translating estimates of relative risk into absolute risk for a meaningful time period’* – may not have been relevant for all studies in our sample
