# Supplementary material for: A review found inadequate reporting of case–control studies of risk factors for pancreatic cancer
Source: J Clin Epidemiol. 2021 May;133:32–42. doi: 10.1016/j.jclinepi.2020.12.020 (PMC8168827; doi:10.1016/j.jclinepi.2020.12.020)
Supplement: Appendix C [file mmc4.pdf]

	STROBE item number		STROBE item reported in sufficient detail to be reproduced	STROBE item reported BUT NOT in sufficient detail to be	STROBE item NOT reported at all	UNSURE (about the information reported)	Not applicable	Page No/Table No
**Title and abstract**	**1a**	Did the authors indicate the study's design with a commonly used term in the title or the abstract?						
	**1b**	Did the authors provide in the abstract an informative and balanced summary of what was done?						
		Did the authors provide in the abstract an informative and balanced summary of what was found?						
**Methods**	**4**	Did the authors present key elements of study design early in the paper?						
	**5**	**Did the authors describe:**						
		The study setting? *Information about setting includes recruitment sites or sources e.g., electoral roll, outpatient clinic, cancer registry, or tertiary care centre .*						
		The study locations? *For example: Information about location may refer to the countries, towns, hospitals or practices where the investigation took place.*						
	**5**	**The relevant dates:**						
		The period of recruitment?						
		The period of exposure?						
		The period of data-collection?						
	**6a**	**Did the authors give:**						
		The eligibility criteria for cases?						
		The ***source*** of cases? *For example a cancer registry or hospital records*						

	The **methods** of case ascertainment?						
	The rationale for the choice of cases?						
	**Did the authors give:**						
	The eligibility criteria for controls?						
	The ***source*** of controls**?** *For example hospital records*						
	The **methods** of control selection?						
	The rationale for the choice of controls?						
**6b**	**For matched studies, did the authors give:**						
	The matching criteria?						
	The number of controls per case?						
**7**	**Did the authors clearly define**						
	All the outcomes?						
	All exposures evaluated?						
	All potential confounders?						
**8**	**For** cases **for each variable of interest did the author give:**						
	Sources of data?						
	Details of methods of assessment (measurement)?						
	**For** controls **for each variable of interest did the author give:**						
	Sources of data?						
	Details of methods of assessment (measurement)?						
**9**	Did the authors describe any efforts to address potential sources of bias?						
**10**	Did the authors explain how the study size was arrived at?						
**11**	Did the authors explain how quantitative variables were handled in the analyses?						
	**If applicable, did the authors describe:**						
	Which groupings were chosen?						
	Why groupings were chosen?						
**12a**	**Did the authors describe:**						
	All statistical methods?						
	Statistical methods....used to control for confounding?						
**12b**	**Did the authors describe**						
**12c**	Did the authors explain how missing data were addressed?						
**12d**	If applicable, did the authors explain how matching of cases and controls was addressed?						

13a	**For cases, did the authors report numbers of individuals at each stage of study:**						
	e.g. numbers potentially eligible?						
	Numbers examined for eligibility?						
	Numbers confirmed eligible?						
	Numbers included in the study?						
	Numbers analysed?						
	**For controls, did the authors report numbers of individuals at each stage of study:**						
	e.g. numbers potentially eligible?						
	Numbers examined for eligibility?						
	Numbers confirmed eligible?						
	Numbers included in the study?						
	Numbers analysed?						
**13b**	For cases, did the authors give reasons for non-participation at each stage?						
**13b**	For controls, did the authors give reasons for non-participation at each stage?						
**13c**	Did the authors include a flow diagram?						
**14a**	**For cases, did the authors give characteristics of study participants:**						
	e.g. demographic, clinical, social details?						
	Information on exposures?						
	Information on potential confounders?						
	**For controls, did the authors give characteristics of study participants:**						
	e.g. demographic, clinical, social details?						
	Information on exposures?						
	Information on potential confounders?						
**14b**	For cases, did the authors indicate number of participants with missing data for each variable of interest?						
	For controls, did the authors indicate number of participants with missing data for each variable of interest?						
**15**	For cases, did the authors report numbers in each exposure category, or summary measures of exposure?						
	For controls, did the authors report numbers in each exposure category, or summary measures of exposure?						

	**16a**	Did the authors give unadjusted estimates?						
		Did the authors report precision for unadjusted estimates? (e.g., 95% confidence interval)?						
		If applicable, did the authors give confounder-adjusted estimates?						
		If applicable, did the authors report precision for confounder-estimates? (e.g., 95% confidence interval)?						
		Did the authors make clear which confounders were adjusted for?						
		Did the authors make clear why confounders were included?						
	**16b**	Did the authors report category boundaries when continuous variables were categorized?						
