# Supplementary material for: A review found inadequate reporting of case–control studies of risk factors for pancreatic cancer
Source: J Clin Epidemiol. 2021 May;133:32–42. doi: 10.1016/j.jclinepi.2020.12.020 (PMC8168827; doi:10.1016/j.jclinepi.2020.12.020)
Supplement: Appendix D [file mmc5.docx]

**Appendix D: Project guidance document summarising guidance from the STROBE Explanation & Elaboration paper**

The guidance is based on the

STROBE Explanation and Elaboration paper: Vandenbroucke, J. P., et al. (2007)

"Strengthening the Reporting of Observational Studies in Epidemiology (STROBE): explanation and elaboration."

PLoS Medicine 4(10): e297.

| **Title and abstract** | | | |
| --- | --- | --- | --- |
|  |  | STROBE Item number | **Text from STROBE E &E** |
|  | 1(*a*) | Did the author indicate the study’s design with a commonly used term in the title or the abstract? | - Readers should be able to easily identify the design that was used from the title or abstract |
|  | 1(*b*) | Did the author provide in the abstract an informative and balanced summary of   - what was done? - what was found? | Typical components include   - A statement of the research question, a short description of methods and results, and a conclusion - Abstracts should summarize key details of studies and should only present information that is provided in the article - **We advise presenting** key results in a numerical form that includes numbers of participants, estimates of associations and appropriate measures of variability and uncertainty (e.g., odds ratios with confidence intervals) - **We regard it insufficient to state** only that an exposure is or is not significantly associated with an outcome. A series of headings pertaining to the background, design, conduct, and analysis of a study may help readers acquire the essential information rapidly |

| **Methods** | | | |
| --- | --- | --- | --- |
| The Methods section **should describe** what was planned and what was done in sufficient detail to allow others to understand the essential aspects of the study, to judge whether the methods were adequate to provide reliable and valid answers, and to assess whether any deviations from the original plan were reasonable | | | |
| Study design | 4 | Did the author present key elements of study design early in the paper? |  |
| Setting | 5 | Did the author describe   - the study setting? - study locations? - periods of recruitment? - period of exposure? - period of data collection? |  |
| Participants | 6(a) | *Case-control studies*  Did the author give…  For cases   - the eligibility criteria? - the source of cases? - the methods of case ascertainment? - the rationale for the choice of cases?   For controls   - the eligibility criteria? - the source of controls? - the methods of control selection - the rationale for the choice of controls? | - **In case-control studies**, the choice of cases and controls is crucial to interpreting the results, and the method of their selection has major implications for study validity. In general, controls should reflect the population from which the cases arose - **We advise** authors to report all eligibility criteria and also to describe the group from which the study population was selected (e.g., the general population of a region or country), and the method of recruitment (e.g., referral or self-selection through advertisement |

| **Methods continued** | | | |
| --- | --- | --- | --- |
|  | 6(b) | *Case-control studies - f*or **matched** studies  Did the author give   - the matching criteria? - the number of controls per case? | - Because matching can be done in various ways, with one or more controls per case, the rationale for the choice of matching variables and the details of the method used s**hould be described** - Commonly used forms of matching are frequency matching (also called group matching) and individual matching - In frequency matching, investigators choose controls so that the distribution of matching variables becomes identical or similar to that of cases - Individual matching involves matching one or several controls to each case. Even apparently simple matching procedures may be poorly reported   For example, authors may state that controls were matched to cases ‘within five years’, or using ‘five year age bands’ |

| **Methods continued** | | | |
| --- | --- | --- | --- |
| Variables | 7 | Did the author clearly define   - all outcomes? - all exposures evaluated? - all potential confounders?     Did the author give diagnostic criteria for outcomes, exposures confounders, if applicable? | - Authors **should define** all variables considered for and included in the analysis, including outcomes, exposures, predictors, potential confounders and potential effect modifiers - Disease outcomes require adequately detailed description of the diagnostic criteria. Clear definitions and steps taken to adhere to them are particularly important for any disease condition of primary interest in the study - For some studies, ‘determinant’ or ‘predictor’ may be appropriate terms for exposure variables and outcomes may be called endpoints’. In multivariable models, authors sometimes use ‘dependent variable’ for an outcome and ‘independent variable’ or ‘explanatory variable’ for exposure and confounding variables. The latter is not precise as it does not distinguish exposures from confounders - If many variables have been measured and included in exploratory analyses in an early discovery phase, **consider providing a list** with details on each variable in an appendix, additional table or separate publication - Finally, **we advise** that authors declare all ‘candidate variables’ considered for statistical analysis, rather than selectively reporting only those included in the final models (see also item 16a) |

| **Methods continued…** | | | |
| --- | --- | --- | --- |
| Data sources/ measurement | 8* | For each variable of interest, did the author give   - sources of data? - details of methods of assessment (i.e. measurement)?     For cases and controls | - It is **helpful….if authors report** the findings of any studies of the validity or reliability of assessments or measurements, including details of the reference standard that was used. Rather than simply citing validation studies - **We advise** that authors give the estimated validity or reliability, which can then be used for measurement error adjustment or sensitivity analyses (see items 12e and 17) - In addition, **it is important to know** if groups being compared differed with respect to the way in which the data were collected |
| Bias | 9 | Did the author describe any efforts to address potential sources of bias? | - **It is important for a reader to know** what measures were taken during the conduct of a study to reduce the potential of bias - At the stage of reporting, **we recommend** that authors always assess the likelihood of relevant biases. Specifically, the direction and magnitude of bias **should be discussed** and, if possible, estimated |

| **Methods continued…** | | | |
| --- | --- | --- | --- |
| Study size | 10 | Did the author explain how the study size was arrived at? | - **We encourage investigators to report** pertinent formal sample size calculations if they were done. In other situations they **should indicate** the considerations that determined the study size - **Do not bother readers with** post hoc justifications for study size or retrospective power calculations |
| Quantitative variables | 11 | Did the author explain how quantitative variables were handled in the analyses?  If applicable, did the author describe   - which groupings were chosen? - why the groupings were chosen? | - **We advise** that authors explain why and how they grouped quantitative data, including the number of categories, the cut-points, and category mean or median values. **Whenever data are reported in tabular form**, the counts of cases, controls, persons at risk, person-time at risk, etc. **should be given** for each category - Tables **should not consist solely** of effect-measure estimates or results of model fitting. Investigators might model an exposure as continuous in order to retain all the information. In making this choice, one needs to consider the nature of the relationship of the exposure to the outcome. As it may be wrong to assume a linear relation automatically, possible departures from linearity should be investigated - **Authors could mention** alternative models they explored during analyses (e.g., using log transformation, quadratic terms or spline functions). Several methods exist for fitting a non-linear relation between the exposure and outcome. Also, **it may be informative** to present both continuous and grouped analyses for a quantitative exposure of prime interest |

| **Methods continued…** | | | |
| --- | --- | --- | --- |
| Statistical methods | 12(a) | Did the author describe all statistical methods, including those used to control for confounding? | - When a study is reported, **authors should tell readers** whether particular analyses were suggested by data inspection. Even though the distinction between pre-specified and exploratory analyses may sometimes be blurred, authors should clarify reasons for particular analyses - Analysts **should fully describe** specific procedures for variable selection and not only present results from the final model - If model comparisons are made to narrow down a list of potential confounders for inclusion in a final model, **this process should be described** - It is **helpful to tell readers** if one or two covariates are responsible for a great deal of the apparent confounding in a data analysis. Other statistical analyses such as imputation procedures, data transformation, and calculations of attributable risks **should also be described**. Nonstandard or novel approaches should be **referenced** and the statistical software used reported - **As a guiding principle, we advise** statistical methods be described ‘‘with enough detail to enable a knowledgeable reader with access to the original data to verify the reported results’’ |

| **Methods continued…** | | | |
| --- | --- | --- | --- |
| Statistical methods | 12(c) | Explain how missing data were addressed | - **We advise** that authors report the number of missing values for each variable of interest (exposures, outcomes, confounders) and for each step in the analysis - **Authors should give** reasons for missing values if possible, and indicate how many individuals were excluded because of missing data when describing the flow of participants through the study (see also item 13) - For analyses that account for missing data, **authors should describe** the nature of the analysis (e.g., multiple imputation) and the assumptions that were made (e.g., missing at random) |
| Statistical methods | 12(d) | If applicable, did the author explain how matching of cases and controls was addressed? | - To allow readers to judge whether the matched design was appropriately taken into account in the analysis, **we recommend** that authors describe in detail what statistical methods were used to analyse the data. If taking the matching into account does have little effect on the estimates, authors may choose to present an unmatched analysis |

| **Results continued** | | | |
| --- | --- | --- | --- |
| Participants | 13(a)* | Did the author report numbers of individuals at each stage of study?   - numbers potentially eligible? - numbers examined for eligibility? - numbers confirmed eligible? - numbers included in the study? - numbers analysed?   For cases and controls | - Investigators should **give an account of** the   numbers of individuals considered at each stage of recruiting study participants, from the choice of a target population to the inclusion of participants’ data in the analysis   - Depending on the type of study, **this may include** the number of individuals considered to be potentially eligible, the number assessed for eligibility, the number found to be eligible, the number included in the study, the number examined, the number followed up and the number included in the analysis - In case-control studies, we advise that authors describe the flow of participants separately for case and control groups - Controls can sometimes be selected from several sources…in this case, **we recommend** a separate account of the numbers of participants for each type of control group |

| **Results continued…** | | | |
| --- | --- | --- | --- |
| Participants | 13(b)* | Did the author give reasons for non-participation at each stage?  For cases and controls | - **Explaining the reasons** why people no longer participated in a study or why they were excluded from statistical analyses helps readers judge whether the study population was representative of the target population and whether bias was possibly introduced |
| Participants | 13(c)* | Did the author present…a flow diagram?  For cases and controls | - The diagram **may usefully include** the main results, such as the number of events for the primary outcome - While **we recommend the use of a flow diagram**, particularly for complex observational studies, we do not propose a specific format for the diagram |
| Descriptive data | 14(a)* | Did the author give characteristics of study participants (e.g. demographic, clinical, social)?  information on exposures?  potential confounders?  For cases and controls | - **Readers need descriptions** of study participants and their exposures to judge the generalisability of the findings - Information about potential confounders, including whether and how they were measured, influences judgments about study validity - **In case-control studies** potential confounders cannot be judged by comparing cases and controls. Control persons represent the source population and will usually be different from the cases in many respects - **In case-control studies** the equivalent of comparing exposed and non-exposed for the presence of potential confounders (as is done in cohorts) can be achieved by exploring the source population of the cases: if the control group is large enough and represents the source population, exposed and unexposed controls can be compared for potential confounders |
| **Results continued…** | | | |
| Descriptive data | 14(b)* | Did the author indicate number of participants with missing data for each variable of interest?  For cases and controls | - **Authors should tell readers** amounts of missing data for exposures, potential confounders, and other important   characteristics of patients (see also item 12c)   - **We advise** authors to use their tables and figures to enumerate amounts of missing data |
| Outcomes data | 15* | *Case-control study*  Did the author report   - numbers in each exposure category? - summary measures of exposure?   For cases and controls | - Before addressing the possible association between exposures (risk factors) and outcomes, **authors should** report relevant descriptive data - It **may be possible** and meaningful to present measures of association in the same table that presents the descriptive data (see item 14a) - **For case-control studies**, the focus will be   on reporting exposures separately for cases and controls as frequencies or quantitative summaries   - It **may be helpful** also to tabulate continuous outcomes or exposures in categories, even if the data are not analysed as such |

| **Results continued…** | | | |
| --- | --- | --- | --- |
| Main results | 16(a) | Did the author give   - unadjusted estimates? - if applicable, confounder-adjusted estimates? - and their precision (e.g. 95% confidence interval)?   Did the author make clear   - which confounders were adjusted for? - why they (i.e. confounders) were included? | - In many situations, authors may present the results of   unadjusted or minimally adjusted analyses and those from fully adjusted analyses.  **We advise** giving the unadjusted  analyses together with the main data, for example the number of cases and controls that were exposed or not   - For adjusted analyses, **report the number** of persons in the analysis, as this number may differ because of missing values in covariates (see also item 12c). Estimates should be given with confidence intervals - Authors **should explain** all potential confounders considered, and the criteria for excluding or including variables in statistical models - If the decision to include a variable in the model was based on the change in the estimate, it is important to **report what change** was considered sufficiently important to justify its inclusion - If a ‘backward deletion’ or ‘forward inclusion’ strategy was used to select confounders, **explain that process** and give the significance level for rejecting the null hypothesis of no confounding |

| **Results continued…** | | | |
| --- | --- | --- | --- |
|  | 16(b) | Did the author report category boundaries when continuous variables were categorized? | - Categorizing continuous data has several important implications for analysis and also affects the presentation of results - In tables, outcomes **should be given** for each exposure category, for example as counts of persons at risk, person-time at risk, if relevant separately for each group (e.g., cases and controls) - **Details** of the categories used may aid comparison of studies and meta-analysis - As a minimum, **authors should report** the category boundaries; it is helpful also to report the range of the data and the mean or median values within categories |
