# Supplementary material for: A review found inadequate reporting of case–control studies of risk factors for pancreatic cancer
Source: J Clin Epidemiol. 2021 May;133:32–42. doi: 10.1016/j.jclinepi.2020.12.020 (PMC8168827; doi:10.1016/j.jclinepi.2020.12.020)
Supplement: Appendix E [file mmc6.docx]

**Appendix E: List of 47 case-control studies evaluated in this study**

1. Abdelrehim MG, Mahfouz EM, Ewis AA, Seedhom AE, Afifi HM, Shebl FM. Dietary Factors Associated with Pancreatic Cancer Risk in Minia, Egypt: Principal Component Analysis. *Asian Pac J Cancer Prev* 2018; **19**(2): 449-455; e-pub ahead of print 2018/02/27; doi 10.22034/APJCP.2018.19.2.449.

2. Antwi SO, Oberg AL, Shivappa N, Bamlet WR, Chaffee KG, Steck SE *et al.* Pancreatic cancer: associations of inflammatory potential of diet, cigarette smoking and long-standing diabetes. *Carcinogenesis* 2016; **37**(5): 481-490; e-pub ahead of print 2016/02/26; doi 10.1093/carcin/bgw022.

3. Archibugi L, Piciucchi M, Stigliano S, Valente R, Zerboni G, Barucca V *et al.* Exclusive and Combined Use of Statins and Aspirin and the Risk of Pancreatic Cancer: a Case-Control Study. *Sci Rep* 2017; **7**(1): 13024; e-pub ahead of print 2017/10/14; doi 10.1038/s41598-017-13430-z.

4. Azeem K, Horakova D, Tomaskova H, Prochazka V, Shonova O, Martinek A *et al.* Evaluation of Dietary Habits in the Study of Pancreatic Cancer. *Klin Onkol* 2016; **29**(3): 196-203; e-pub ahead of print 2016/06/15; doi 10.14735/amko2016196.

5. Ben Q, Liu J, Wang W, Guo F, Yao W, Zhong J *et al.* Association between ABO blood types and sporadic pancreatic neuroendocrine tumors in the Chinese Han population. *Oncotarget* 2017; **8**(33): 54799-54808; e-pub ahead of print 2017/09/15; doi 10.18632/oncotarget.18592.

6. Ben Q, Zhong J, Fei J, Chen H, Yv L, Tan J *et al.* Risk Factors for Sporadic Pancreatic Neuroendocrine Tumors: A Case-Control Study. *Sci Rep* 2016; **6**: 36073; e-pub ahead of print 2016/10/27; doi 10.1038/srep36073.

7. Cui Y, Shu XO, Li HL, Yang G, Wen W, Gao YT *et al.* Prospective study of urinary prostaglandin E2 metabolite and pancreatic cancer risk. *Int J Cancer* 2017; **141**(12): 2423-2429; e-pub ahead of print 2017/08/18; doi 10.1002/ijc.31007.

8. Fan X, Alekseyenko AV, Wu J, Peters BA, Jacobs EJ, Gapstur SM *et al.* Human oral microbiome and prospective risk for pancreatic cancer: a population-based nested case-control study. *Gut* 2018; **67**(1): 120-127; e-pub ahead of print 2016/11/02; doi 10.1136/gutjnl-2016-312580.

9. Gomez-Rubio P, Zock JP, Rava M, Marquez M, Sharp L, Hidalgo M *et al.* Reduced risk of pancreatic cancer associated with asthma and nasal allergies. *Gut* 2017; **66**(2): 314-322; e-pub ahead of print 2015/12/03; doi 10.1136/gutjnl-2015-310442.

10. Hicks B, Friis S, Pottegard A. Use of proton pump inhibitors and risk of pancreatic cancer. *Pharmacoepidemiol Drug Saf* 2018; **27**(8): 926-930; e-pub ahead of print 2018/06/21; doi 10.1002/pds.4576.

11. Huang J, Zagai U, Hallmans G, Nyren O, Engstrand L, Stolzenberg-Solomon R *et al.* Helicobacter pylori infection, chronic corpus atrophic gastritis and pancreatic cancer risk in the European Prospective Investigation into Cancer and Nutrition (EPIC) cohort: A nested case-control study. *Int J Cancer* 2017; **140**(8): 1727-1735; e-pub ahead of print 2016/12/30; doi 10.1002/ijc.30590.

12. Huang JY, Butler LM, Midttun O, Koh WP, Ueland PM, Wang R *et al.* Serum B6 vitamers (pyridoxal 5'-phosphate, pyridoxal, and 4-pyridoxic acid) and pancreatic cancer risk: two nested case-control studies in Asian populations. *Cancer Causes Control* 2016; **27**(12): 1447-1456; e-pub ahead of print 2016/11/11; doi 10.1007/s10552-016-0822-6.

13. Huang JY, Butler LM, Midttun O, Ulvik A, Wang R, Jin A *et al.* A prospective evaluation of serum kynurenine metabolites and risk of pancreatic cancer. *PLoS One* 2018; **13**(5): e0196465; e-pub ahead of print 2018/05/08; doi 10.1371/journal.pone.0196465.

14. Katagiri R, Goto A, Nakagawa T, Nishiumi S, Kobayashi T, Hidaka A *et al.* Increased Levels of Branched-Chain Amino Acid Associated With Increased Risk of Pancreatic Cancer in a Prospective Case-Control Study of a Large Cohort. *Gastroenterology* 2018; **155**(5): 1474-1482; e-pub ahead of print 2018/08/05; doi 10.1053/j.gastro.2018.07.033.

15. Kearns MD, Boursi B, Yang YX. Proton pump inhibitors on pancreatic cancer risk and survival. *Cancer Epidemiol* 2017; **46**: 80-84; e-pub ahead of print 2017/01/06; doi 10.1016/j.canep.2016.12.006.

16. Keum N, Ha KH, Bao Y, Chung MJ, Kim HC, Giovannucci EL. Long-term patterns of fasting blood glucose levels and pancreatic cancer incidence. *Cancer Causes Control* 2018; **29**(1): 135-142; e-pub ahead of print 2017/12/05; doi 10.1007/s10552-017-0988-6.

17. Khalaf N, Yuan C, Hamada T, Cao Y, Babic A, Morales-Oyarvide V *et al.* Regular Use of Aspirin or Non-Aspirin Nonsteroidal Anti-Inflammatory Drugs Is Not Associated With Risk of Incident Pancreatic Cancer in Two Large Cohort Studies. *Gastroenterology* 2018; **154**(5): 1380-1390 e-pub ahead of print 2017/12/13; doi 10.1053/j.gastro.2017.12.001.

18. Kho PF, Fawcett J, Fritschi L, Risch H, Webb PM, Whiteman DC *et al.* Nonsteroidal anti-inflammatory drugs, statins, and pancreatic cancer risk: a population-based case-control study. *Cancer Causes Control* 2016; **27**(12): 1457-1464; e-pub ahead of print 2016/11/07; doi 10.1007/s10552-016-0824-4.

19. Lener MR, Scott RJ, Wiechowska-Kozlowska A, Serrano-Fernandez P, Baszuk P, Jaworska-Bieniek K *et al.* Serum Concentrations of Selenium and Copper in Patients Diagnosed with Pancreatic Cancer. *Cancer Res Treat* 2016; **48**(3): 1056-1064; e-pub ahead of print 2016/01/05; doi 10.4143/crt.2015.282.

20. Li X, Xu H, Gao P. ABO Blood Group and Diabetes Mellitus Influence the Risk for Pancreatic Cancer in a Population from China. *Med Sci Monit* 2018; **24**: 9392-9398; e-pub ahead of print 2018/12/26; doi 10.12659/MSM.913769.

21. Lucas AL, Bosetti C, Boffetta P, Negri E, Tavani A, Serafini M *et al.* Dietary total antioxidant capacity and pancreatic cancer risk: an Italian case-control study. *British journal of cancer* 2016; **115**(1): 102-107; e-pub ahead of print 2016/05/14; doi 10.1038/bjc.2016.114.

22. Lujan-Barroso L, Zhang W, Olson SH, Gao YT, Yu H, Baghurst PA *et al.* Menstrual and Reproductive Factors, Hormone Use, and Risk of Pancreatic Cancer: Analysis From the International Pancreatic Cancer Case-Control Consortium (PanC4). *Pancreas* 2016; **45**(10): 1401-1410; e-pub ahead of print 2016/10/18; doi 10.1097/MPA.0000000000000635.

23. Mandilaras V, Bouganim N, Yin H, Asselah J, Azoulay L. The use of drugs acting on the renin-angiotensin system and the incidence of pancreatic cancer. *British journal of cancer* 2017; **116**(1): 103-108; e-pub ahead of print 2016/11/16; doi 10.1038/bjc.2016.375.

24. Marley AR, Fan H, Hoyt ML, Anderson KE, Zhang J. Intake of methyl-related nutrients and risk of pancreatic cancer in a population-based case-control study in Minnesota. *Eur J Clin Nutr* 2018; **72**(8): 1128-1135; e-pub ahead of print 2018/06/16; doi 10.1038/s41430-018-0228-5.

25. Masoudi S, Momayez Sanat Z, Mahmud Saleh A, Nozari N, Ghamarzad N, Pourshams A. Menstrual and Reproductive Factors and Risk of Pancreatic Cancer in Women. *Middle East J Dig Dis* 2017; **9**(3): 146-149; e-pub ahead of print 2017/09/13; doi 10.15171/mejdd.2017.65.

26. Matejcic M, Lesueur F, Biessy C, Renault AL, Mebirouk N, Yammine S *et al.* Circulating plasma phospholipid fatty acids and risk of pancreatic cancer in a large European cohort. *Int J Cancer* 2018; **143**(10): 2437-2448; e-pub ahead of print 2018/08/16; doi 10.1002/ijc.31797.

27. McWilliams RR, Maisonneuve P, Bamlet WR, Petersen GM, Li D, Risch HA *et al.* Risk Factors for Early-Onset and Very-Early-Onset Pancreatic Adenocarcinoma: A Pancreatic Cancer Case-Control Consortium (PanC4) Analysis. *Pancreas* 2016; **45**(2): 311-316; e-pub ahead of print 2015/12/10; doi 10.1097/mpa.0000000000000392.

28. Mei QX, Huang CL, Luo SZ, Zhang XM, Zeng Y, Lu YY. Characterization of the duodenal bacterial microbiota in patients with pancreatic head cancer vs. healthy controls. *Pancreatology* 2018; **18**(4): 438-445; e-pub ahead of print 2018/04/15; doi 10.1016/j.pan.2018.03.005.

29. Mohamadkhani A, Pourshams A, Viti J, Cellai F, Mortazavi K, Sharafkhah M *et al.* Pancreatic Cancer is Associated with Peripheral Leukocyte Oxidative DNA Damage. *Asian Pac J Cancer Prev* 2017; **18**(5): 1349-1355; e-pub ahead of print 2017/06/15; doi 10.22034/APJCP.2017.18.5.1349.

30. Molina-Montes E, Gomez-Rubio P, Marquez M, Rava M, Lohr M, Michalski CW *et al.* Risk of pancreatic cancer associated with family history of cancer and other medical conditions by accounting for smoking among relatives. *Int J Epidemiol* 2018; **47**(2): 473-483; e-pub ahead of print 2018/01/13; doi 10.1093/ije/dyx269.

31. Mueller AM, Meier CR, Jick SS, Schneider C. The Potential of Glycemic Control and Body Weight Change as Early Markers for Pancreatic Cancer in Patients With Long-standing Diabetes Mellitus: A Case-Control Study. *Pancreas* 2018; **47**(7): 807-815; e-pub ahead of print 2018/07/06; doi 10.1097/MPA.0000000000001085.

32. Nakagawa T, Kobayashi T, Nishiumi S, Hidaka A, Yamaji T, Sawada N *et al.* Metabolome analysis for pancreatic cancer risk in nested case-control study: Japan Public Health Center-based prospective Study. *Cancer Sci* 2018; **109**(5): 1672-1681; e-pub ahead of print 2018/03/27; doi 10.1111/cas.13573.

33. Nogueira LM, Newton CC, Pollak M, Silverman DT, Albanes D, Mannisto S *et al.* Serum C-peptide, Total and High Molecular Weight Adiponectin, and Pancreatic Cancer: Do Associations Differ by Smoking? *Cancer Epidemiol Biomarkers Prev* 2017; **26**(6): 914-922; e-pub ahead of print 2017/01/18; doi 10.1158/1055-9965.EPI-16-0891.

34. Olson SH, Satagopan J, Xu Y, Ling L, Leong S, Orlow I *et al.* The oral microbiota in patients with pancreatic cancer, patients with IPMNs, and controls: a pilot study. *Cancer Causes Control* 2017; **28**(9): 959-969; e-pub ahead of print 2017/08/02; doi 10.1007/s10552-017-0933-8.

35. Onyeaghala G, Nelson HH, Thyagarajan B, Linabery AM, Panoskaltsis-Mortari A, Gross M *et al.* Soluble MICA is elevated in pancreatic cancer: Results from a population based case-control study. *Mol Carcinog* 2017; **56**(9): 2158-2164; e-pub ahead of print 2017/05/05; doi 10.1002/mc.22667.

36. Pelucchi C, Rosato V, Bracci PM, Li D, Neale RE, Lucenteforte E *et al.* Dietary acrylamide and the risk of pancreatic cancer in the International Pancreatic Cancer Case-Control Consortium (PanC4). *Ann Oncol* 2017; **28**(2): 408-414; e-pub ahead of print 2016/11/12; doi 10.1093/annonc/mdw618.

37. Peng YC, Lin CL, Hsu WY, Lu IT, Yeh HZ, Chang CS *et al.* Proton Pump Inhibitor Use is Associated With Risk of Pancreatic Cancer: A Nested Case-Control Study. *Dose Response* 2018; **16**(4): 1559325818803283; e-pub ahead of print 2018/10/06; doi 10.1177/1559325818803283.

38. Risch HA, Lu L, Streicher SA, Wang J, Zhang W, Ni Q *et al.* Aspirin Use and Reduced Risk of Pancreatic Cancer. *Cancer Epidemiol Biomarkers Prev* 2017; **26**(1): 68-74; e-pub ahead of print 2016/12/22; doi 10.1158/1055-9965.EPI-16-0508.

39. Schulte A, Pandeya N, Fawcett J, Fritschi L, Klein K, Risch HA *et al.* Association between family cancer history and risk of pancreatic cancer. *Cancer Epidemiol* 2016; **45**: 145-150; e-pub ahead of print 2016/11/05; doi 10.1016/j.canep.2016.10.005.

40. Shakeri R, Kamangar F, Mohamadnejad M, Tabrizi R, Zamani F, Mohamadkhani A *et al.* Opium use, cigarette smoking, and alcohol consumption in relation to pancreatic cancer. *Medicine (Baltimore)* 2016; **95**(28): e3922; e-pub ahead of print 2016/07/20; doi 10.1097/MD.0000000000003922.

41. Shu X, Zheng W, Yu D, Li HL, Lan Q, Yang G *et al.* Prospective metabolomics study identifies potential novel blood metabolites associated with pancreatic cancer risk. *Int J Cancer* 2018; **143**(9): 2161-2167; e-pub ahead of print 2018/05/03; doi 10.1002/ijc.31574.

42. Sun Y, Wu J, Cai H, Wang S, Liu Q, Blot WJ *et al.* A prospective study of autoantibodies to Ezrin and pancreatic cancer risk. *Cancer Causes Control* 2016; **27**(6): 831-835; e-pub ahead of print 2016/05/06; doi 10.1007/s10552-016-0757-y.

43. Valente R, Hayes AJ, Haugvik SP, Hedenstrom P, Siuka D, Korsaeth E *et al.* Risk and protective factors for the occurrence of sporadic pancreatic endocrine neoplasms. *Endocr Relat Cancer* 2017; **24**(8): 405-414; e-pub ahead of print 2017/06/02; doi 10.1530/ERC-17-0040.

44. van Duijnhoven FJB, Jenab M, Hveem K, Siersema PD, Fedirko V, Duell EJ *et al.* Circulating concentrations of vitamin D in relation to pancreatic cancer risk in European populations. *Int J Cancer* 2018; **142**(6): 1189-1201; e-pub ahead of print 2017/11/09; doi 10.1002/ijc.31146.

45. White DL, Hoogeveen RC, Chen L, Richardson P, Ravishankar M, Shah P *et al.* A prospective study of soluble receptor for advanced glycation end products and adipokines in association with pancreatic cancer in postmenopausal women. *Cancer Med* 2018; **7**(5): 2180-2191; e-pub ahead of print 2018/03/25; doi 10.1002/cam4.1426.

46. Yallew W, Bamlet WR, Oberg AL, Anderson KE, Olson JE, Sinha R *et al.* Association between Alcohol Consumption, Folate Intake, and Risk of Pancreatic Cancer: A Case-Control Study. *Nutrients* 2017; **9**(5); e-pub ahead of print 2017/05/05; doi 10.3390/nu9050448.

47. Zheng Z, Zheng R, He Y, Sun X, Wang N, Chen T *et al.* Risk Factors for Pancreatic Cancer in China: A Multicenter Case-Control Study. *J Epidemiol* 2016; **26**(2): 64-70; e-pub ahead of print 2015/10/07; doi 10.2188/jea.JE20140148.
